# Clinical Profile and Outcome of Resistant Gram-Negative Infections in a Pediatric Intensive Care Unit: A Retrospective Study

**DOI:** 10.7759/cureus.102212

**Published:** 2026-01-24

**Authors:** Shadi Shweiyat, Nasser Banikhaled, Yacoub M Haddadin, Hala Alhajaj, Shaden Alsaryrah, Anees Hjazeen, Alia F Al-Khlaifat

**Affiliations:** 1 Pediatric Critical Care, Queen Rania Al Abdullah Hospital for Children, Amman, JOR; 2 Pediatric Infectious Diseases, Queen Rania Al Abdullah Hospital for Children, Amman, JOR; 3 Pediatric Intensive Care Unit, Queen Rania Al Abdullah Hospital for Children, Amman, JOR; 4 Microbiology, Jordanian Royal Medical Services, Amman, JOR; 5 Community Health Nursing and Biostatistics, Jordanian Royal Medical Services, Amman, JOR; 6 Pediatric Infectious Diseases, Jordanian Royal Medical Services, Amman, JOR

**Keywords:** antibiotics therapy, gram-negative multidrug-resistant (mdr), klebsiella, pediatric intensive care unit, pediatrics patients

## Abstract

Aim

Healthcare-associated infections (HCAIs) cause increased morbidity and mortality in intensive care units. The aim of this study is to evaluate the characteristics of resistant, gram-negative infections in a pediatric intensive care unit (PICU) and identify clinical and microbiological risk factors associated with mortality. Comprehending these factors is essential for enhancing patient outcomes and directing treatment interventions in the PICU.

Materials and methods

Data were collected from pediatric patients admitted to the PICU at Queen Rania Al Abdullah Hospital for Children, Amman, Jordan, between January 2020 and December 2024. Patients with gram-negative multidrug-resistant organism (MDRO) infections receiving antimicrobial treatment were included. Demographic data, antimicrobial drug exposures, and outcomes such as mortality were recorded.

Results

A mortality rate of 32.3% was observed for the study sample. Gram-negative organisms such as *Klebsiella* and *Escherichia coli* showed high resistance to multiple antibiotics.

Conclusion

Age is a critical factor in predicting survival outcomes in PICUs, highlighting the necessity for customized interventions based on patient demographics and clinical features. The increasing prevalence of multidrug-resistant bacteria in PICUs is a pressing concern that demands immediate attention.

## Introduction

Healthcare-associated infections (HCAIs) are a significant global problem in intensive care units. Infection incidence in pediatric and neonatal intensive care units (PICU and NICU) varies from 6% to 12% and 10% to 25%, respectively [1]. These infections can lead to increased morbidity, mortality, and healthcare costs. Therefore, it is crucial to implement effective infection control measures in these specialized units to prevent and control HCAIs. Additionally, HCAIs are the most frequent adverse event in children admitted to intensive care units (ICUs) [2]. ICU patients are more likely to be exposed to multidrug-resistant organisms (MDROs) and HCAIs, primarily due to the increased vulnerability of sick patients, use of invasive devices, and use of antimicrobials. Children are even more susceptible to these infections because their immune systems are not fully developed, and during times of acute illness, both innate and adaptive immunity are compromised [3].

Resistant Gram-negative bacteria are a major cause of HCAIs in pediatric intensive care, leading to serious infections and increased risks for critically ill children. These bacteria are rod-shaped and identified by their pink/red appearance on a Gram stain. Their rising prevalence complicates treatment and highlights the need for effective detection and infection control.

The emergence of antibiotic resistance in microorganisms, leading to nosocomial infections, is becoming an increasingly major concern. It is advisable to conduct investigations at preset intervals to establish the distribution of infectious agents and the rates of antibiotic resistance. The choice of suitable antibiotics for treatment will depend on the identification of the causative agent of the infection and the incidence of antibiotic resistance.

This study focused on identifying infectious agents in the PICU at Queen Rania Al Abdullah Hospital for Children, Amman, Jordan, from January 2020 to December 2024. The study aimed to determine the prevalence of antibiotic resistance among these infectious agents. Furthermore, we aimed to evaluate the clinical and microbiological risk factors for 30-day mortality associated with MDRO infections in patients in the PICU.

The study results offer critical insights into the prevalence and trends of antibiotic resistance in the PICU at Queen Rania Al Abdullah Hospital for Children. Additionally, understanding the clinical and microbiological risk factors associated with mortality can help inform strategies for improving patient outcomes and guiding appropriate treatment interventions.

## Materials and methods

Methods

This retrospective study was conducted on PICU patients at Queen Rania Al Abdullah Hospital for Children between January 2020 and December 2024. Only patients with active and symptomatic infections receiving antimicrobial treatment were included.

Demographic data

Patient characteristics, including age, gender, co-morbid illness, clinical course (total length of stay), and outcome, were recorded.

Definitions

The study was conducted using the Centers for Disease Control and Prevention (CDC)’s HCAI case definitions. The inclusion criteria were only those infections presenting and identified >48 hours after admission to the ICU as ICU-acquired [4]. Isolated bacteria were defined as MDROs if resistance to at least one antibiotic agent in three or more different antibiotic classes was shown [5].

Fungal infections were excluded. The distinction between colonization and infection at the site of infection was made by evaluating clinical criteria, such as the presence of fever and the adequacy of organ perfusion [6]. All the included patients met the criteria for infection.

Identification of isolates

The isolates were recognized based on colony morphology on MacConkey agar, blood agar, and chocolate agar. Here, the Gram stain of the smear was made from the isolated colonies. The VITEK® 2 (bioMérieux, Marcy-l'Étoile, France) GN 21341 card on the VITEK® 2 Compact Automated System was used for species-level identification of Gram-negative bacilli.

Antimicrobial susceptibility test

The antimicrobial sensitivity was determined using the VITEK® 2 Compact Automated System, with VITEK® 2 AST-N417 and VITEK® 2 AST-XN20 cards.

Statistical analysis

The Mann-Whitney U and Chi-square tests, with a p-value < 0.05, were used to describe categorical variables, verify the significance of differences among subgroups, and identify any statistical significance. The Mann-Whitney U was used for median differences in the age variable between survivors and non-survivors. Chi-square tests were used for proportion differences in gender and underlying disease between the two groups. A p-value < 0.05 was deemed statistically significant, and IBM SPSS Statistics for Windows, Version 28 (Released 2021; IBM Corp., Armonk, NY, USA) was used.

## Results

A total of 723 specimens were processed, and growth was obtained in 493 specimens. Among these, 62 isolates were identified as Gram-negative bacilli. The patients who were admitted to the PICU during the study period included 58.1% (n = 36) males and 41.9% (n = 26) females. The median age of the patients was six months (range, 1-168 months). Death during the hospital stay was reported for 32.3% (20/62) of the patients. Common underlying causes of admission in the PICU included postoperative (30.5%), neurological (21%), metabolic (12.9%), cardiac (9.7%), renal (8.1%), gastrointestinal (8.1%), hematological (6.5%), and chest (3.2%) diseases.

*Klebsiella pneumoniae* was the most common Gram-negative multidrug-resistant (MDR) bacterium, making up 38.7% of the total. Of these, only 7% showed sensitivity to cephalosporins, while 64% were sensitive to carbapenems. Additionally, 57% were sensitive to aminoglycosides, 21% to piperacillin/tazobactam and ciprofloxacin, and all 14 cases (100%) to colistin. The median hospital stay for *Klebsiella* was 18 days, with a mortality rate of 14.5%. For *Escherichia coli*, out of a total of 12 cases (19.4%), 20% showed sensitivity to cephalosporins, 80% to carbapenems and amikacin, and 60% to piperacillin/tazobactam and ciprofloxacin; all 12 cases (100%) were sensitive to colistin. The median hospital stay for *E. coli* was five days, with a mortality rate of 25%.

As for *Acinetobacter*, out of 10 cases (16.1%), none showed sensitivity to cephalosporins, carbapenems, amikacin, ciprofloxacin, or piperacillin/tazobactam. However, all 10 were sensitive to colistin. The average length of stay in the hospital for *Acinetobacter* was 36 days, and the death rate was 30%.

For *Pseudomonas*, out of a total of seven cases (11.3%), none showed sensitivity to cephalosporins, while 33% were sensitive to carbapenems and 67% were sensitive to aminoglycosides. Additionally, all seven cases were sensitive to piperacillin/tazobactam, colistin, and ciprofloxacin. The median hospital stay for *Pseudomonas* was three days, with a mortality rate of 28.6%.

Only one of the six cases (23.1%) of *Enterobacter aerogenes* was sensitive to cephalosporins. However, 75% of those cases were sensitive to carbapenems, and all of them were sensitive to piperacillin/tazobactam, colistin, and ciprofloxacin. The median hospital stay for *E. aerogenes* was 26 days, with a mortality rate of 33.3%. 

For *Stenotrophomonas*, out of a total of five cases (8.1%), none were sensitive to cephalosporins, carbapenems, ciprofloxacin, amikacin, or piperacillin/tazobactam. However, all five cases (100%) were sensitive to trimethoprim/sulfamethoxazole, and 67% were sensitive to colistin. The median hospital stay for *Stenotrophomonas* was five days, with a mortality rate of 20%. Table 1 shows the antibiotic susceptibility patterns, median hospital stay (in days), and mortality rate for Gram-negative pathogens.

**Table 1:** Gram-negative pathogens and their antibiotic susceptibility patterns, hospital stay median (days), and mortality. CEP: cephalosporins; CAR: carbapenems; AMI: amikacin; PIP: piperacillin/tazobactam; COL: colistin; CIP: ciprofloxacin; TRI: trimethoprim/sulfamethoxazole; HS. Med: hospital stay median; MOR: mortality; *E. coli*: *Escherichia coli*; *E. aerogenes*: *Enterobacter aerogenes*

Gram-negative pathogens	n (%)	CEP	CAR	AMI	PIP	COL	CIP	TRI	HS. Med (days)	MOR (rate%)
Klebsiella	24	7	64	57	21	100	21	-	18	14.5
E. coli	12	20	80	80	60	100	60	-	5	25
Acinetobacter	10	0	0	0	0	100	0	-	36	30
Pseudomonas	7	0	33	67	100	100	33	-	3	28.6
E. aerogenes	6	0	75	100	100	100	100	-	26	33.3
Stenotrophomonas	5	0	0	0	0	67	0	100	5	20

The distribution of agents by culture specimens (Table 2) reveals that peripheral blood was the most common source, accounting for 29 isolates (46.8%), followed by discharge/pus with 15 isolates (24.2%) and catheter samples with 10 isolates (16%), while both urine and tracheal aspirate cultures (TACs) each contributed four isolates (6.5%).

**Table 2:** Distribution of agents by culture specimens. TAC: tracheal aspirate culture

Specimen type	Total (n, %)
Peripheral blood	29 (46.8%)
Discharge/Pus	15 (24.2%)
Catheter-associated infections	10 (16%)
Urine	4 (6.5%)
TAC	4 (6.5%)

The study analyzed the main characteristics of the patients in the PICU and divided them into survivors and non-survivors days after admission. Out of the 62 patients studied, 42 survived, while 20 did not (Table 3).

**Table 3:** Main characteristics of the studied patients subdivided into survivors and non-survivors 30 days after admission to the PICU. * denotes Mann-Whitney U test; # denotes Chi-square (χ²). dF: degrees of freedom; PICU: pediatric intensive care unit

Groups	Survivors n = 42	Non-survivors n = 20	dF	Test value		p-value
^*^Age median (months)	1	30	-	Z = 2.29		<0.001
#Gender			1	χ² = 3.96		0.060
Female	14	12	-			
Male	28	8				
^#^Underlying disease			7	χ² = 4.69		0.698
Cardiac	5	1	-			
Chest	1	1				
Gastrointestinal	4	1				
Hematological	4	0				
Metabolic	4	4				
Neurological	9	4				
Post-operative	12	7				
Renal	3	2				

The results indicated that there was a median difference in patients’ ages based on cohort groups, revealing that the non-surviving patients were older than the survivors, with a median age of 30 vs. 1.0 months, as determined by the Mann-Whitney U test (p < 0.001). However, neither bacterial type, MDR, nor underlying causes showed a significant association with survival or non-survival, as determined by a Chi-square test (p = 0.06). Female gender and metabolic or postoperative disease were more common among non-survivors, but these differences did not reach statistical significance. Hematological disease was observed only in survivors, but was not statistically significant (p = 0.0698).

## Discussion

MDR is considered a significant global public health issue that leads to higher rates of mortality and morbidity due to treatment ineffectiveness [7]. A list of the most MDR bacteria was released by the World Health Organization. These bacteria pose a serious threat to human health and require specialized research in order to create a new supply of antibiotics [8].

Among Gram-negative bacteria, *K. pneumoniae* was the most commonly detected organism in this study (24%). This result aligns with findings from Ergül et al. [9], who also identified *K. pneumoniae* as the predominant *Enterobacteriaceae* species, followed by *Serratia* spp. and reported *E. coli* as notably rare (1.2%). Similarly, Banik et al. highlighted that, among Gram-negative pathogens, *Enterobacteriaceae* collectively accounted for the highest number of sepsis cases, with *Klebsiella* species and *E. coli* being the principal contributors [10]. This opportunistic pathogen is a major source of infections related to medical care, primarily affecting patients with compromised immune systems [11]. The high prevalence of MDR *K. pneumoniae* among PICU patients is likely due to their immunocompromised state. Infections with MDR *K. pneumoniae* have been associated with increased morbidity and mortality in low- and middle-income countries [12].

Among Gram-negative bacteria, *K. pneumoniae* and *E. coli* were the most common (36/62), with 64% and 80% carbapenem susceptibility, respectively. A review of Gram-negative bacteria indicates that aminoglycosides and carbapenems show greater susceptibility, suggesting that they should be prioritized in our hospital for treating infections caused by these bacteria.

Nosocomial infections in critically ill patients are frequently caused by *Acinetobacter baumannii* [13]. In our study, *Acinetobacter* isolates demonstrated no sensitivity to cephalosporins, carbapenems, amikacin, ciprofloxacin, or piperacillin/tazobactam, with colistin being the most effective agent. These results are consistent with those of Ergül et al. [9], who reported 100% resistance to carbapenems and 90% to aminoglycosides among *Acinetobacter* spp., while observing no resistance to colistin. Tosi et al. previously considered carbapenems as first-line agents for severe infections caused by *Acinetobacter*; however, their use is increasingly restricted in many regions due to the rising prevalence of resistance [14]. Collectively, these findings highlight the growing challenge of MDR *Acinetobacter* in critical care and reinforce the importance of colistin as an essential therapeutic option. Further research is needed to determine the most effective treatment strategies for *Acinetobacter* infections in this light.

Out of all 62 cases, there were 20 deaths, yielding a mortality rate of 32.3%, far from that suggested by the Pediatric Health Information System database in the USA, which estimates a mortality rate of 14.4% in the PICU [15]. The sample size in this case may be too small to allow accurate comparison with a larger database. Additionally, mortality rates can vary based on factors such as region, hospital resources, and patient population. However, higher rates have been detected in other developing countries, ranging between 10% and 53.6%. In contrast, very low mortality rates have been reported in developed countries [16].

Regarding Table 2, the majority of culture specimens were collected from peripheral blood, accounting for 46.8% of the total. Discharge/pus samples were the second most common type, representing 24.2% of the total distribution. Catheter-associated infections, urine samples, and TACs made up the remaining 23% of the distribution. These results indicate a diverse range of specimen types collected for analysis.

Overall, the findings highlight the need for vigilant monitoring and targeted infection prevention strategies, particularly for peripheral blood and postoperative infections, which together comprise over three-quarters of the identified cases. This data underscores the importance of implementing strict infection control measures in healthcare settings to prevent the spread of these infections. It also emphasizes the need for ongoing surveillance and intervention to reduce the burden of device-related infections in critically ill patients. These results emphasize the importance of implementing strict infection control measures in healthcare settings to reduce the risk of device-related infections. Additionally, ongoing surveillance and education on proper catheter care may help decrease the incidence of urinary tract infections and tracheal aspirate infections in this population.

The median age of the surviving patients was one month, significantly lower than that of the non-survivors, with a median age of 30 months, as determined by the Mann-Whitney U test (p < 0.01). Gender did not show a significant association with survival outcome (p = 0.060). Although many reports suggested that no correlation was observed between age and mortality [17-19], in our study, the surviving patients were significantly younger than the non-survivors. This finding raises questions about the impact of age on susceptibility to MDR bacteria and the ability to combat infections in pediatric patients. Further investigation is needed to determine if age-related factors, such as immune system development or comorbidities, play a role in the outcomes of pediatric patients in intensive care settings. Additionally, exploring potential interventions or treatment strategies that may be more effective in younger patients could lead to improved outcomes and better management of MDR infections in this vulnerable population. While gender may have been close to significance in our study, it is important to consider the limitations of the sample size and potential confounding variables that could have affected the results. Additionally, previous studies have shown that predictors of poor outcomes in patients with MDRO infections can vary depending on the population and methodology used [20].

The current study primarily investigated the association between MDRO infections and mortality outcomes in the PICU. Post-operative cases accounted for the highest percentage of admissions, followed by neurological, chest, renal, cardiac, hematological, gastrointestinal, and metabolic diseases. Interestingly, the underlying cause of admission did not show a significant association with survival outcome. This finding suggests that, while underlying causes play a crucial role in patient management and treatment, they may not be the primary determinant of survival in the PICU.

It is important to note that the sample size for non-survivors (n = 20) is significantly smaller than the sample size for survivors (n = 42), which may influence the Chi-square test results and potentially obscure any true associations between bacterial type, MDR, or underlying causes and survival. Furthermore, other factors not considered in this study may influence patient survival outcomes in the PICU. We excluded any patients who had no cultures collected. While culture-negative sepsis is a recognized problem, inclusion of patients with no collected cultures could introduce bias and affect the validity of the study results. The current study sought to ensure that the data collected accurately reflect the population being studied.

## Conclusions

The results of this study shed light on the impact of age, gender, bacterial species, underlying causes, antibiotic susceptibility patterns, mortality rates, and length of hospital stay on pediatric patients with MDRO infections in the PICU. While age emerged as a significant predictor of survival, other factors, such as gender, bacterial species, and underlying causes, did not show a significant association with patient outcome. By understanding the complex interplay of these factors, healthcare providers can tailor treatment strategies, optimize care, and improve outcomes for PICU patients. This study underscores the importance of personalized care, multidisciplinary approaches, and evidence-based interventions in managing MDRO infections in the pediatric population.
